# Observations on Anti-Predator Defense Behavior in Feral Horses in Venezuela

**DOI:** 10.3390/ani16121826

**Published:** 2026-06-12

**Authors:** Lucy Rees, Emily Kieson

**Affiliations:** 1 Pottokas en Piornal, 10615 Piornal, Spain; 2 Equine International, Boston, MA 02115, USA; ekieson@equineintl.org

**Keywords:** horse, anti-predator defense, puma, alarm, social cohesion

## Abstract

Apart from vigilance and flight, anti-predator defense behavior in horses has not been well documented despite its importance during natural selection. In this study, observations of a feral herd (around 140) of Venezuelan horses sympatric with puma and jaguar divided such defense into precaution and reaction. Group living and the avoidance of danger areas are precautionary measures enhanced by the stallion’s vigilance and his actions to keep small foals with the band. Reactions to perceived threats comprise communication of alarm; bunching, or cohesion, as a primary response; massed flight following self-organizing principles; and reassembly of bands. Stallions usually initiated this behavioral process. Stallions’ initial reactions to perceived threats were “investigation”, “move away”, “run away”, and “stampede”, and resulting herd behavior was categorized into 27 responses. Data analysis through Observation Oriented Modeling indicated that each category of initial stallion response to perceived threats was associated with a recurring pattern of subsequent herd behavior. Prominent behaviors enhanced cohesion and synchrony, as well as velocity and direction matching. A fourth observed category was the cohesive “run to band” of a startled outlying member, in which the individual’s alarm might transmit to the band or the band’s calm transmit to the individual. The results emphasize the importance of communication, social cohesion, and synchronous action in times of perceived threats, their continuous practice during maintenance activities, and the social needs and understanding management of domestic horses.

## Simple Summary

This study of feral Venezuelan horses living amongst puma and jaguar categorized defense tactics into precautionary and reactive behaviors. Stallions, in addition to keeping vigil, contributed to band cohesion and returning foals to the band and assumed a primary role in assessing potential danger; they communicated their conclusions in specific behaviors that initiated specific collective band responses. Acute alarm provoked band cohesion and flight, which followed the algorithm of self-organized mass movement seen in other flocks, herds, and shoals. When alarm spreads to other bands, they cohere into a stampeding herd. The study highlights the importance of social cohesion and communication in horses, with implications for understanding their behavior and welfare.

## 1. Introduction

This study addresses an important but neglected area of equine ethology: the behavioral responses of horses (*Equus ferus caballus*) to predator threats. Predation has exerted a constant pressure on equid evolution since the Miocene [1], and the present *Equus* evolved among a formidable array of Pleistocene predators. Its evolving morphological specializations for rapid take-off and sustained speed indicate that detection and flight became the horse’s major form of anti-predator defense. The tendency to flee when alarmed is a persistent behavioral trait of horses, even domestic ones that have not been exposed to predation for many generations [2]. The generalization and conservation of this response indicate a deep-rooted survival strategy adapted to situations demanding defense against any type of predator or perceived threat [3].

Flight is energy-consuming, and other behavioral adaptations like risk assessment, avoidance of high-risk situations, social interaction, and communication are likely to affect decisions to flee [4]. However, the role of predation in shaping horse behavior has not been well examined, partly for lack of evidence. The subject receives scant mention in equine ethograms [5,6,7,8,9,10] beyond the fact that both feral and domestic horses take avoidance action in the face of perceived threats [6,11,12].

In all social prey animals, group living is seen to offer anti-predator protection [13] by reducing individual predation risk and facilitating shared vigilance [4], communication of alarm [14], and fast grouping to provide dilution and predator eye confusion effects [15]. In herds, flocks, and shoals, the characteristics of massed flight emerge from the self-organizing three-part algorithm commonly seen in collective movements: cohesion, alignment of direction and velocity (synchrony), and non-collision [16,17,18]. The impact of these general principles on horse anti-predator defense and social dynamics has not been established beyond some basic observations.

Feral horses live in relatively stable family bands consisting of a stallion (sometimes two), a few mares, and their offspring up to maturity [6,10,11]. The stallion’s main role is to maintain the integrity of the band and to protect it against predators and other males [8]. In keeping with this defensive role, stallions spend more time in vigilance than mares [11,19]. Perceiving a potential threat, horses raise their heads quickly—a possible communication signal. Feist and McCullough [6] and Waring [10] showed head and neck positions corresponding to vigilance and alarm; however, correlations of head position to physiological measures are difficult to interpret, for terms such as “alarm”, “alert”, “vigilant”, “head-raising”, “neck elevation”, and “stress” are often used without distinguishing definitions or illustrations [20,21]. Also, the flight that may follow acute alarm is not documented.

Horses may need to adjust their behaviors according to local predator species and environmental context. In the face of puma or bear, increased wariness and tendency to flight are more likely than attack [6], though horses do attack smaller predators. Berger and Rudman [22] saw mustang mares and stallions attack coyote interested in newborn foals or cleansings (placenta or amniotic membranes). There are anecdotal accounts of stallions and mares attacking wolves, and mares grouping around foals to protect them [23], but flight remains a prevalent option.

Anti-predator defense has become of particular interest since the spread of both wolves and rewilding projects in Europe. Voigtländer-Schnabel et al. [24] reviewed the wolf predation literature in relation to suitable management of such projects. They commented on the lack of detailed observations of anti-predator defense behavior in horses—how it develops, the roles of social interaction and communication, or the relevance of predator danger to the horse’s social organization and dynamics. Feh et al. [25] saw wolf attacks on kulan (*Equus hemionus hemionus*), which either fled or turned to attack the wolves. They concluded that the social structure of kulan groups reflects predator danger, and that the same might be true of horses.

In the absence of direct observational studies of horse anti-predator defense behavior, some experimental investigations have been made. On seeing a model puma, feral mustang stallions showed alarm [5] and stepped forward assertively; stallions with foals persisted longer than stallions without foals. They showed no such strong reactions to a model springhorn. Janczarek et al. [26,27] investigated the reactions of domestic horses to predator calls, showing differences according to breed and call, especially in grouping behavior. Janicka et al. [28] used a far wider variety of 40 sounds, also with domestic horses, concluding that novelty, predictability, and animal origin provoked mild alarm irrespective of whether the animal was a predator or not. Predator odors or horse blood were shown not to frighten domestic horses, but these did increase vigilance and reaction to other sudden stimuli [29]. Thus, whether horses have innate reactions to predators or simply to a variety of novel or startling stimuli is still an open question. Habituation, sensitization, and relaxation of defense behavior in domestic horses selected for calm behavior are to be expected [3,4,12]. Direct observation of feral horses living and breeding successfully among predators is necessary.

This observational study arose during group ethology courses on feral horse behavior in the llanos of Venezuela. The horses were known to be preyed on by large felines. Alarms and flights were frequent, and visibility across the flat, open savannah was exceptional. As the uniqueness of this situation became clear, observations became directed to behaviors presumably related to the presence of predators, with the consideration of the possible relevance of these behaviors to social dynamics. As investigations proceeded, two questions became salient:

Do flight patterns correspond to self-organizing collective movements?

What, if any, is the stallion’s special role in influencing the behavior of others?

The degenerating socio-political climate in Venezuela curtailed research before the suggested hypothesis could be more rigorously tested with adequate technology. This is a pilot study on a neglected area.

## 2. Materials and Methods

In the llanos of Venezuela, the great flood plain of the Orinoco, feral horses, known as cimarrons, formerly roamed in abundance. Originating mainly from the herds on La Hispañola established by the conquistadors [30], their numbers were swelled by escapes during the Bolivian wars (1810–1820) and subsequently diminished by ranching activities. The population studied here was perhaps the last remaining true feral herd and sympatric with puma (*Puma concolor)* and jaguar (*Panthera onca*).

**Figure 1:**
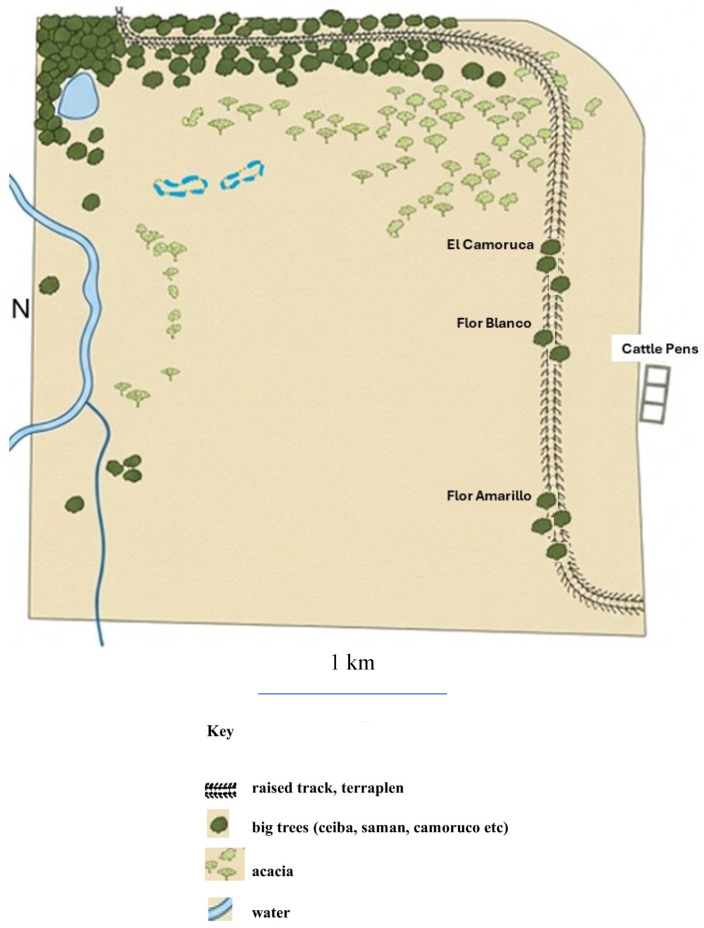
Sketch map of the cimarrons’ enclosure.

Hato Los Camorucos is a 11,000 ha cattle ranch near Mantecal, Apure, in the southern llanos. A boundary fence erected in the 1920s enclosed about 300 cimarrons. Half the herd was removed to breed ranch horses; the other half, about 150, was left without any management, except for the removal of 4–10 bachelors in the years before 2007 when the managed herd did not produce enough colts to castrate for ranch work. In 1990, the cimarrons’ range was reduced to a 1200 ha enclosure. The area is a flat savannah and mainly treeless, except towards the east where acacia scrub gives way to forest. Satellite images do not accurately reflect the topography and vegetation at the time of the observational study, so a sketch map shows the key features of the study site (Figure 1).

Male jaguar in the llanos average 80 kg and females 50 kg [31]. Jaguar may be capable of killing small adult horses (average weight 300 kg), but they do not leave the cover of the forest. Puma, which are much smaller (50 kg male, 25 kg female [29]) do hunt on open savannah. Their main diet is capybara [29] (*Hydrochoerus hydrochaeris*), which are abundant, but they also kill foals. Puma are often visible before dawn; their trail and scat are easily found, but the population size is unknown. The adjoining 80,000 ha ranch, Hato El Frío, was a nature reserve until 2011; in Los Camorucos, puma are not killed unless they attack the calves near the ranch house (one female was killed in 2011), while jaguar are not killed at all, so both populations remain at near-natural levels.

There are two six-month seasons in the llanos, with a hot, dry summer from November to the end of April, followed by rainy winter. After a month’s rain, the rivers burst their banks, flooding the entire llanos until October. In the horses’ area, only the northeastern forest and a raised track or terraplen running west–east remain above the water, creating more favorable conditions for clumps or copses of large trees to grow. The horses live in the flood, grazing underwater. During the study periods, they shared the area with Brahma cattle, which were removed before the rains’ start.

### 2.1. Population

**Figure 2:**
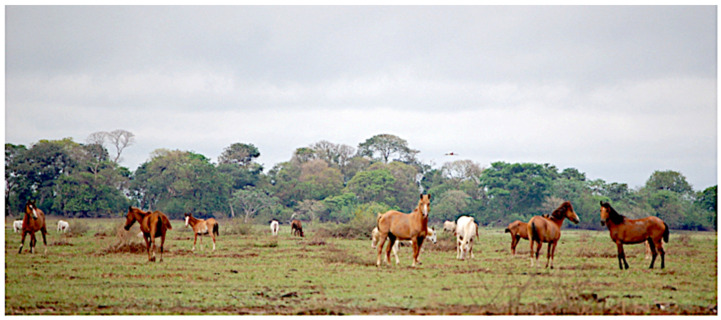
The stallion Guapo in alarm posture. His mares stop grazing and begin to bunch together. The white mare ignoring him belongs to another band; we note the short tails. Behind them are the terraplen and trees of the copse Flor Amarillo.

The horses were visited by all ten members of the ethology course, two instructors and eight students, throughout April each year from 2007 to 2011. During 2007–2010, the population remained stable at 136–143 individuals over a year old, comprising 12–14 stallions, 20–25 bachelors, and 56–62 breeding mares, with the remainder being sub-adults or young mares. The age of youngsters can usually be estimated by tail length, but the constant submergence in winter floods rots the end of the tail, meaning that all horses had tails as short as foals’ (see Figure 2).

Mares first foaled at four or five years old and subsequently in alternate years; in late March–April, only one mare ever foaled in two successive years. During April 2008–2010, an estimated 17–20% of the previous year’s foals did not survive to be yearlings; without the tail length to positively identify yearlings, these figures are best estimates. During those years, 22 foals definitely disappeared and 17 of their bodies were found. Seven were located near dead mares and five more, also located near dead mares, showed signs of puma kill: lacerations of the head and neck, viscera torn out, and signs of dragging or attempts at concealment [32]. One foal died of an ear infection (ticks and maggots). Further foal mortality occurred during the rain if foals were born late.

Typical causes of adult death are endemic trypanosomiasis and outbreaks of infectious anemia (Dr. Vargas, veterinarian and ranch owner, pers. comm.), both causing gradual debilitation. Horses usually die in the winter months, and their flesh disappears in the floods, leaving only osteological remains. The teeth showed that most horses lived at least 25 years unless other causes hastened death. During 2007–2010, one bachelor died of a Pitium infection, one stallion died of tetanus, and four skeletons had broken teeth and/or abscessed mandibula that probably contributed to death.

In 2011, the population suffered major disruptions. An unusual piroplasmosis outbreak killed a quarter of the population. At the beginning of April, 29 bodies (20 mares, 6 males, 3 foals) were found and five mature stallions had disappeared; throughout April, two mares, one foal, and two bachelors died. Deaths are listed in Supplementary Materials. Also in 2011, five entire male mules escaped from the ranch and joined the cimarrons. They harassed the mares by chasing and attempting to rape them; they also charged at and dislodged stallions mounting mares.

All individuals were identified photographically each year. Feral horse groupings are normally of two types [6,10,11,33]. Natal bands, which are relatively stable over years, comprise mares and their offspring with a stallion (sometimes two). At sexual maturity, youngsters disperse from their natal bands. Young males group in rather less stable bachelor bands until they form natal bands themselves. Stallions protect their natal bands against intruding males, and natal bands are usually found separately. However, the Camoruco stallions showed unusual mutual tolerance, and bands often fused to form large groups or one large herd, with peaceful intermingling of members. Also present were mixed-sex groups of youngsters, estimated to be around 2 years old (i.e., around natal dispersal), which were unstable groups that attached themselves temporarily to natal bands, so that natal band sizes might fluctuate from day to day even though the core adults remained constant. A few mares also drifted from band to band throughout the years of observation.

Adult horses were assigned to natal bands as core members or transients. Stallions were named with their initials following the order of their first identification to help students in discrimination and evaluation; many mares were also named. However, instant identification was sometimes impossible due to band fusion and the prevalence of chestnut horses whose socks, if present, were invisible in the scrub; later photographic identification was often possible except when horses were moving fast. Records of natal bands and their stallions are given in Supplementary Materials.

### 2.2. Methods

During each April from 2007 to 2011, the horses were watched daily from 05:30 to 10:00 (96 days, 3 overnights, 412 h). The observer group compromised L. Rees, another instructor, and 8 students, all trained in observation and familiar with the horses. The group of 10 observers arrived by truck and, often splitting into smaller groups, could advance to about 100 m from the horses. Observations were made with binoculars or the naked eye by L.R. and at least two other trained observers to ensure consistency of observed behavioral recording.

**Figure 3:**
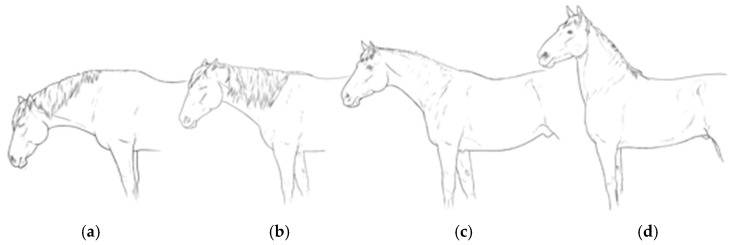
Head and neck positions. Left to right: (**a**) asleep, eyes closed, ears turned to side, whole head below the level of the withers, suspended on the nuchal ligament; (**b**) drowsy, neck horizontal, ears flickering; (**c**) vigilant, half of head level with withers, ears pricked forward, gaze forwards; (**d**) alarm posture, muzzle at or above withers, eyes wide open, neck fully elevated, tension in all muscles, tail a little elevated [6,10].

Students were trained to distinguish head and neck positions related to arousal state, as shown in Figure 3, and to recognize rapid head-raising into the alarm position that also captures the attention of other horses.

With the aim of clarifying the details of reactions to alarm at perceived threats, subsequent actions were coded by hand by L.R., using abbreviations from the ethogram developed during preliminary observations during 2007 and 2008, as shown in Table 1.

This enabled a standardized registration of the reactions of the first horse to be alarmed; of the behavior of the band and of other bands; and whether the alarm subsided or resulted in movement, in flight away from the danger, or in stampede (that is, all visible horses galloping together). Additional observers, always present, corroborated the sequence of behavioral transitions, contributing to identification of or calling attention to the reactions of bands distant from the immediate action. Finalized notes excluded events where the complete sequence from initial alarm to post-event calm was neither seen nor confirmed by all present, giving a total of 77 analyzable events out of a total of over 200. Also excluded were brief alarm postures followed by return to grazing or resting. The well-documented sequences were recorded mainly in 2010 and 2011 when experienced additional observers were present.

Permission to observe the horses and horse behavior was given by local authorities, and no additional ethical permissions were required for observational research according to local governing laws.

### 2.3. Statistical Analysis/Observation Oriented Modeling (OOM)

Pattern analysis was conducted using Observation Oriented Modeling (OOM) version 2 (Integrated Knowledge Systems Inc., Scottsdale, AZ, USA; 2016), which evaluates the extent to which observed data conform to a defined pattern using Percent Correct Classification (PCC) and a randomization-based chance value (c-value) rather than relying on population–parameter inference. While PCC values are often accompanied by a visual representation of the raw data and a percentage (%), the randomization trials are not displayed graphically in the concatenated orderings output; their results are summarized by the reported c-value, which is expressed similar to *p*-values. OOM permits both a priori and post hoc pattern analysis derived directly from the observed data [34,35,36]. In the present study, OOM was used as a descriptive approach to examine pattern consistency in coded behavioral sequences. It was selected because the dataset consisted of categorized event sequences rather than continuous repeated measures analyzed at the level of individual identities. The large totals reported in the OOM output reflect the number of coded binary orderings contributed by each event sequence, not independent behavioral events. Previous studies have also applied OOM to equine behavioral data [35,37]. In the OOM framework, observations are coded into explicitly defined units and observed classification accuracy is compared with classification accuracies obtained from randomized datasets. The OOM manual identifies PCC as the primary summary index of classification accuracy, with the c-value representing the proportion of randomized trials that equal or exceed the observed PCC. OOM also reports classification accuracy at the level of complete cases. In the present study, Percent Correct Classification (PCC) refers to the percentage of all coded observations that matched the defined pattern, whereas Percent Correct Classified Cases refers to the percentage of full event sequences that matched the defined pattern across all included orderings.

For this study, OOM pattern analysis using concatenated orderings was performed for four event categories: run away, move away, investigation, and stampede. These categories were assigned at the event level on the basis of the observed overall behavioral outcome of the sequence: investigation events involved assessment without band flight; move away events included the stallion moving away at walk or trot; run away events involved band galloping over a shorter distance; and stampede events involved whole-herd flight over a substantially greater distance. These analyses were used to examine whether the coded behavioral sequences within each event category showed greater pattern consistency than expected under randomization. The analyses were conducted in OOM using the settings Missing Values = Omitted from Totals, Randomization Method = within Cases, and 1000 randomized trials for each pattern. For each event category, coded event sequences were entered into OOM as 27 binary orderings. The observed PCC was calculated. OOM then generated 1000 randomized within-case versions of the dataset and recalculated PCC for each randomized trial. The c-value was computed as the proportion of randomized PCC values equal to or greater than the observed PCC. Observed sequences were therefore compared with randomized versions generated within the OOM software (https://oom.solutions/) at the level of the individual event sequence.

Each event sequence was represented as 27 binary orderings, corresponding to the coded presence (1) or absence (0) of specific behavioral elements across the sequence, including the first action, second action, third action, reaction of the focal band, and reaction of other bands. 

The first action category included orientating in alarm, move away, run away, run to band, and run toward danger. The second action category included step towards, run away, run to band, run toward danger, dither, and retrieve. The third action category included stand and stare, turn away, and move away. Focal band responses included none, bunch, synchronize, move away, parallel, run toward danger, run to band, and run away. Responses of other bands included none, bunch together, synchronize with first band, stampede, and piñas.

The number of complete event sequences included in each OOM analysis was: run away, *n* = 10; move away, *n* = 13; investigation, *n* = 20; and stampede, *n* = 11. Because each complete event contributed one coded value to each of the 27 binary orderings, the corresponding total numbers of OOM observations were 270, 351, 540, and 297, respectively.

For each analysis, OOM reports both classification accuracy across all coded observations and classification accuracy across complete cases. In the present manuscript, PCC and c-values are reported descriptively as indices of pattern consistency relative to randomized sequences. To avoid overstating inference, the OOM results are interpreted as evidence that the observed coded sequences were more consistent with the defined patterns than randomized sequences rather than as direct tests of causation or population-level behavioral rules. In addition, because these event sequences were drawn from repeated observations of the same population and the defined patterns were examined within the present dataset, the OOM outputs are interpreted cautiously as descriptive analyses of pattern consistency rather than as independent confirmatory tests. Potential non-independence among events and the possibility of dataset-specific fit should therefore be kept in mind when interpreting the results.

## 3. Results

**Figure 4:**
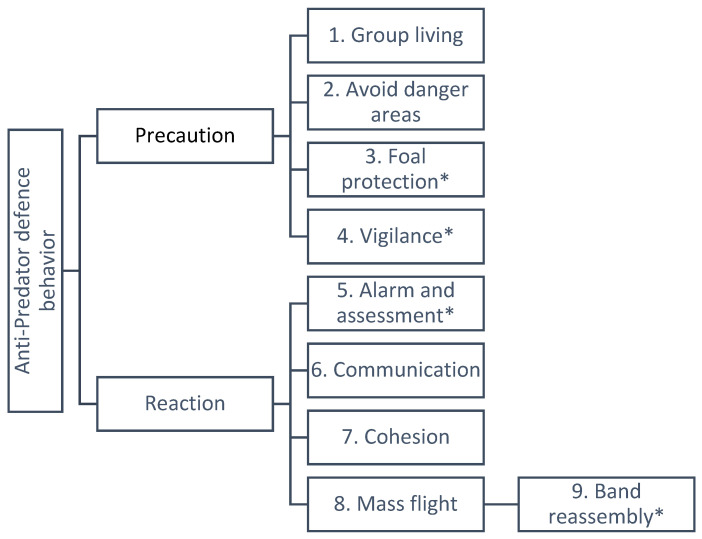
Overview of precautionary and reactive components of alarm and flight behavior observed in the Camoruco cimarrons. * Areas in which the stallion plays a key though not exclusive role.

Since alarm and flight are the horse’s preferred response to large predators [6], their high frequency in the studied population was assumed to be a response to the ever-present danger of puma attack. Although no direct attacks were seen, it is possible that the horses perceived felines when observers did not. Alarm and flight form a part of the array of anti-predator defense behaviors adopted by the population. These fell into two main categories, precaution and reaction, comprising nine elements in all (Figure 4):

### 3.1. Precautionary, Proactive Behavior

1. Social grouping: At dawn, on 48% of days (n = 96), there was one single herd; on other days, there was a large group with several satellite bands, usually bachelors or novice stallion bands. As the day drew on, these large associations broke up into component bands, but band fission and fusion recurred constantly throughout the day, and the herd reformed at night. Natal band mares numbered from one, in the case of a novice stallion, to eight or nine in the larger bands with old stallions. In the herd or in large groups, bands tended to be found in particular orders or configurations, though whether this was the stallions’ choice or the mares’ is unknown. Stallions with in-season mares separated their bands from the main herd (except Careto, whose band was always fused with others until he sickened with tetanus); so too did novice stallions. The savannah is so flat that even when bands were separated, they usually remained in visual contact, facilitating communication of alarm between, as well as within, bands.

Bachelor bands, usually five to seven strong, often hovered around the edges of groups but could also be found far from others in less frequented areas of the savannah. Two stallions, Elegante and Dorado, allowed up to four bachelors (not their sons) in their natal bands, often playfighting with them. These vigorous games, observed also in bachelor bands, included rearing, neck-wrestling, and kneeling in response to bites behind the elbow but did not result in aggression. Bachelors were never seen challenging or fighting a resident stallion, and the largest natal bands were those of the oldest stallions.

Only once was a horse seen alone: a primiparous mare that became separated from her yearling during a stampede and stood alone for two days until he rejoined her. No solitary bachelors or displaced stallions were seen.

2. Avoidance of danger areas: Trees allow surprise attack by puma and jaguar and also harbor vampire bats (*Desmodus rotundus*). The horses never entered the forest, and on the rare occasions (four in all) that bands grazed the lush pasture round the drying lagoon, they were noticeably more vigilant and easily startled than usual. They were most often found in the center of the area, where there were no bushes, grazing and resting at temperatures up to 40 °C; at nightfall, they crossed the terraplen and spent the night together near the cattle pens, an area of poor grazing but devoid of cover. From 2007 to 2010, there was no trace of them in the copses on the terraplen, though cattle often rest and die there. In 2011, during the piroplasmosis epidemic, three dead mares and foals were found in one copse; the bodies of the foals showed kill signs more typical of jaguar than puma [31], with the back of the skull removed and the brains licked out. One sick mare rested in the shade there and died the next day.

Although in other years the herd crossed or circumvented the terraplen well before dawn to reach better grazing areas, in 2011, they lingered by the pens until after daybreak. They were nervous about crossing between the copses, tending to hesitate, bunch together, and cross at a run, with stallions leading most bunches. That year, there were many prints of a puma with cubs near the copses, and they were sighted early one morning.

3. Foal retrieval by stallions: Although mares were attentive in keeping newborn foals in close proximity, after the post-partum estrus stallions played a more important role in ensuring foals stayed with the band. Stallions retrieved sleeping foals left behind as the band drifted along while grazing or went back to return them to the band on multiple-band marches, using nose nudges and/or the head-down herding position. Some stallions were more attentive to this duty than others: Amiguete was seen retrieving foals on eight occasions during April 2010, but Jotero never did. Mares were not seen to retrieve foals in these situations, although they called foals when alarmed.

Stampedes can be lethal for foals, which stop galloping once no longer alongside their mothers. On three occasions, foals were found abandoned after stampedes. Out of a total of 34 flight events, stallions turned back at the height of a stampede four times to retrieve foals left behind, and three times to accompany slow mares with new foals. During the piroplasmosis outbreak, stallions on two occasions slowed during flight to accompany sick mares without foals; in both cases, the stallion and the mare were noted for their habitual close proximity and interchange of affiliative behavior.

4. Vigilance: Stallions often acted as sentinels, standing in vigilant position (Figure 3) for minutes on end (53 min on one occasion), especially at dawn and dusk; at midday, vigilance was relaxed. Mares acted as sentinels if a stallion was eating, and in two-stallion bands, the pair alternated irregularly.

While grazing, stallions raised their heads to scan more often than mares did, averaging 2.63 head raises per 5 min compared with 1.00 for mares. Because these observations were repeated within the same population and were not analyzed at the level of independently resolved individuals, this comparison is presented descriptively only, but it corresponds well with more rigorous investigation (20).

5. Alarm: On detecting a possible threat, a horse rapidly orientated towards it (O, Table 1) if necessary, raising the head abruptly to position d in Figure 2, the alarm posture (A), with ears pricked towards the threat. Tension in all muscles was observed particularly in the back, raising the tail and shortening stride length. The horse might take one or two jerky steps forwards or sideways and/or snort loudly.

When individuals could be identified, the first behaviors indicating alarm were expressed by stallions 36 times, ignored by the rest of the band once; mares 12 times, ignored three times; and a youngster once (n = 49). It was not possible to reliably determine whether the observed tendency for stallions to initiate alarm reflected a population-wide pattern or disproportionate contribution by a subset of individuals. Although many stallions expressing alarm behavior were identified with confidence, the large number of stallions represented across years, and occasional uncertainty in distinguishing very similar individuals, prevented robust analysis of individual contributions.

Visual, auditory, or vibrational causes of alarm were identified; olfactory ones may also exist but could not be determined at the time.

(a) Visual: Unidentified or brusque movement, often more than 1 km away, especially near or in trees or bushes; for example, an outlying horse suddenly galloping towards the band. Indirect or transmitted alarm, such as the sight of other bands bunching or fleeing. Movement caused 40% of alarms (n = 89). The sight of other bands bunching caused another 7%.
(b) Vaqueros: Mounted vaqueros inspected or rounded up the cattle about once a week. The sight of them, or even just one, always alarmed the horses, who usually stampeded. Vaqueros provoked 18% of 89 alarms.
(c) Auditory: Rustles in bushes (movement not always seen, but the horses startled suddenly when passing near bushes); shouts from invisible vaqueros; the sound of invisible galloping horses; and, on one occasion, an earth-moving machine starting up. Sounds provoked instant flight rather than orientation and examination and were responsible for 7% of 89 alarms.
(d) Observers: In the open savannah, it was impossible to approach the horses without being detected. The horses gradually became habituated to people on foot, allowing them within 50 m by 2011, but turning a notebook page suddenly would cause alarm. On four occasions, horses grazed towards observers lying down and only became alarmed upon recognizing humans. Observers caused 11% of 89 alarms.
(e) The truck: A pickup brought and fetched the group of observers. In 2007 and half of 2008, the horses stampeded on seeing it from far off, circling to a stop in the central area, downwind from the group of researchers, orienting towards them and lifting their heads to a horizontal position to smell. Later they became habituated, and in 2011, they merely lifted their heads when the truck arrived. At the start of each year’s observation period, reactions were stronger than at the end of the previous year (rebound effect) but died down more quickly. Of 89 alarm events, 8% were caused by the truck.
(f) Cows running: Sleeping calves sometimes woke up suddenly, ran calling for their mothers, and set the cows running. On three occasions, the horses joined in.
(g) Vibration: Earth shaking due to invisible galloping horses on the other side of the terraplen (perceptible to observers, two occasions). The horses ran in one direction and then another; on one occasion, some bands went in one direction and the rest in another.
(h) Miscellaneous unusual events: Among these, a water buffalo, a helicopter, and a machine starting up also caused alarm and short flights.

Assessment of perceived threats: Although sounds, vibrations, or sudden movement nearby might provoke instant flight (17% of 77 events), other threats initiated a varying period of assessment by the stallion. If necessary, he moved to place himself between the mares and the perceived threat, or to stand beside an alarmed mare. Various options then followed.

(a) He might relax into vigilant position, standing staring (SS, maximum observed time 18 min) before resuming other activities. When the horse displayed no alarm after quickly identifying the stimulus and the band did not react, the event was discarded. The initial alarm varied in strength and could not be estimated without precise coding of video records.
(b) He might investigate the threat. All sequences included either standing staring in a vigilant position (SS) or stepping towards it (Mt); the stallion might also displace a few steps laterally, snort, and/or visibly smell the air with distended nostrils and head held horizontally (23.4% of 77 events).
(c) He might vacillate, showing a variable sequence of more than nine rapid behavioral changes comprising steps towards (Mt), turn away (Ta), move away (Ma), reorientation (O), and standing staring (SS), typical of approach–avoidance conflict behavior. Displacements were of one or a few steps only. The sequence contained repeated elements and terminated in the horse walking away in four sequences (5.2%, n = 77), with one by a mare.
(d) He might turn and move away at a walk or trot (Ma), or turn and run (Ra). Movement away might be punctuated or terminated by the stallion’s reorientation towards the threat and standing staring (61.5%, n = 26).

6. Communication: The easily perceived signs of alarm—rapid head-raising and alarm posture—alerted others in the band, who raised their heads in varying degrees of alarm too. Without detailed video analysis, it was difficult to determine whether alarms that appeared to be ignored were not perceived by others, were perceived and rapidly assessed as trivial (as are the alarms of foals), or were caused by events already dismissed as non-threatening. Mules, present only in 2011, gave frequent dramatic alarm signals, accompanied by loud repeated snorts, that were almost always ignored by the horses.

**Figure 5:**
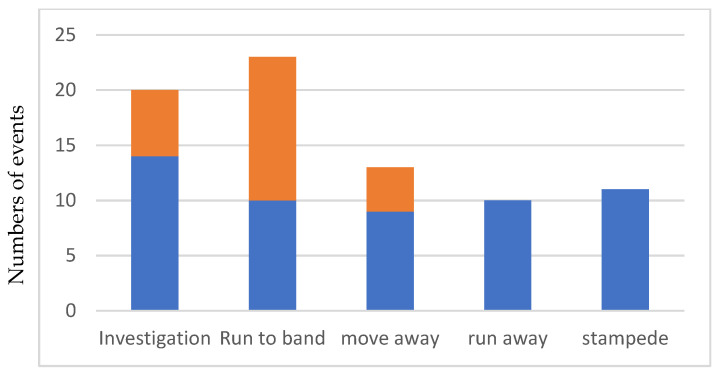
Broad classes of 77 alarm events. 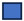 Events in which members of band bunched, i.e., moved to cohere closely together. 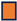 No bunching.

The response to alarms manifests in widely varying ways best considered from the perspective of five classes of final outcome, termed (i) investigation; (ii) run to band; (iii) move away; (iv) run away; and (v) stampede (Figure 5). In all of these, two features are notable:

(a) Sex differences in reactions to mild and/or unidentified threats;
(b) Bunching (cohesion) and movement synchrony shown by band. Both increase with alarm.

(i) Investigation (inv), *n* = 20: Sequences did not include Ra, while 90% included Mt. The stallion interposed himself between the threat and the rest of the band and stepped towards the threat. When a mare gave the alarm, he moved to place himself alongside her or nearer to the threat in all observed cases. Mares moved to bunch together, some 5–7 m behind the stallion, separated only by an individual space, and their youngsters with them, in 70% of cases.

Second stallions, in-band bachelors, or sons lined up in parallel with the stallion and synchronized closely with his actions in 70% of cases; half of these involved all the individuals in bachelor bands. Only once did a (sub-adult) mare do this.

(ii) Run to band, *n* = 23: Sequences included Rtb. An outlying horse, usually some 50 m away from its band, startled and ran towards the band. In five cases (23%), the horse ran to the band before turning to check what had startled it. In ten cases (43.5%), the band bunched together, looking in the direction from whence it came but only walked away twice. Strikingly, when mares were chased by stallions (more usually, mules) and galloped into a band, apparently frightened, the band never reacted by bunching or becoming alarmed. The relatively large number of these events suggests that horses are more prone to alarm when isolated from their bands.
(iii) Move away, *n* = 15: Sequences included the stallion turning and moving away at a walk or trot. Overall, 69.2% began with the sequence described above in investigation, with the mares bunching behind the stallion, though no parallel line-ups were seen.
(iv) Run away, *n* = 10: Sequences included the whole band galloping between about 30 and 200 m. Half (50%) began with the stallion orienting in alarm (OA) before turning to run. In 60% of cases, bands nearby also joined their flight.
(v) Stampede, *n* = 11: Sequences involved the whole herd (once, all but one band) fleeing, usually between 2 and 5 km. Only 27.2% started with one horse orientating in alarm and the band bunching behind the stallion; the remainder started instantaneously, and the band bunched and synchronized their movements when already in flight.

Table 2 gives a breakdown of behavior during recorded events. A more detailed breakdown can be found in the Excel sheet in Supplementary Materials.

7. Bunching, or cohesion: Apart from head-raising, the clearest primary response to alarm or the sight of an alarm posture was bunching, or cohesion, of the mares and youngsters behind the stallion. With a brief look at each other and the stallion, the mares moved together until separated by just under a horse’s length (individual distance), looking towards the disturbance; foals pressed close to their dams’ sides.

In total, 88% of 67 alarm postures provoked bunching; four (one given by a restlessly moving mare) were apparently ignored by others in the band. Four provoked head-raising only. These latter four were responses either to brief reactions from the stallion or, conversely, to long sequences of his vacillations in classic approach/avoidance conflict.

When bands were close or fused and all bunched together in alarm, outlying bands ran to join the mass. As with isolated individuals running towards their own band, isolated bands (more than 100 m from others) running towards and bunching with others either slowed to a walk and ceased to display signs of alarm (30.8%) or provoked flight in the others (69.2%) (N = 26). Other bands might join them until the whole population was in flight and stampeding; in roundups, they merged with the cattle. Sick or lame horses unable to keep up with horse-only stampedes sought and joined a bunch of calm cattle.

In 22% of 23 individual flights to join the band, as well as in 30% of 10 band flights towards other bands, flight paths at first led the horses nearer to the threat before cohering with others. On two occasions, single or small groups of bands forged their way across streams of fast-moving cattle to reach stationary bands on the other side.

8. Band synchrony: Synchrony here is used to mean animals moving together matching direction, velocity, and posture; when two horses are separated only by individual distance, synchrony includes them moving the same legs at the same time. The band synchronized with avoidance action by the stallion, though only males synchronized with investigative behavior. If the stallion turned to flee, the mares and youngsters did the same simultaneously, so that the stallion followed the close-packed band. On four occasions, the stallion Jotero moved to the leading position in his band, never being seen to follow it; other stallions always brought up the rear, except when leading a dawn dash across the terraplen. (Conversely, Jotero never led these runs though others did.)

There were no fixed leaders in either single- or multiple-band flights. As bands joined a stampede, leading positions were usually taken by the speedy two- and three-year-olds, changing constantly. The central mass of mature horses did not always follow the leaders; if it changed direction, so did the leaders. In cattle roundups, vaqueros moved in a parallel line from the north, driving the horses and cattle southwards towards the pens beyond the terraplen at a gallop. As the mixed herd approached the terraplen, the experienced mature horses swerved to the right, thus avoiding being trapped in the pens beyond it. The leaders did not change direction until the main block behind them did so.

Seen from the front, all horses maintained a clear space of about 1 m between themselves and their neighbors, except small foals that stayed pressed to their mothers’ sides. No collisions were seen. The herd sometimes split to avoid bushes but rapidly cohered again.

All observed stampedes, including those not included in the analysis for lack of details, ended up in the central area where there are no bushes. Stampedes provoked by vaqueros rounding up cattle developed in exactly the same way as those provoked by other causes, showing a generalized behavioral defense response to any perceived threat.

Variations: Flights provoked by sounds started instantly, without the stallion investigating the source, and tight band cohesion occurred during the initial flight.

If the stallion stood staring at or investigating the possible threat without turning or moving away, the mares gradually relaxed, spread out and resumed grazing after five to ten minutes. Overall, 37% of 54 noted alarm events did not escalate into flight; probably many more went unnoticed. Where a clear decision was not forthcoming (vacillating), the mares also lost interest.

The stallion might stare, investigate, and assess for minutes before deciding to move away. When walking away, he might turn and stop to stare; in this case, the rest of the band did not stop, but when he turned back to retrieve a slow mare, they also slowed. If the stallion trotted to catch up with the others, they trotted until he walked again. When the stallion Careto was lame with a tetanus-infected wound, his mares startled suddenly and ran, but in circles without leaving his vicinity.

Flight termination: Flight terminated in various ways. When an outlying individual fled into the center of a band, its members may or may not become startled too. Similarly, a band might flee to the main herd, with the same results, or it might flee only a few hundred meters before stopping. A whole-herd stampede covered 2–5 km before their speed dropped to a trot and walk, when the horses would spread out and weave across each other’s paths, neighing and reforming bands.

9. Band reassembly—the “piña”: Some half-hour after the end of 55.6% of whole-herd stampedes (N = 12), many bands formed huddles or “piñas” (pineapple or defensive grouping). The mares, aggressively excluding youngsters and non-band members, stood in a U-shaped formation, with their heads in the central space. The stallion stood across the open end of the U, perpendicular to the breeze, which blew across his body into the U. They remained in this position for 30–60 min. Piñas were not seen after shorter flights where band unity was not lost, except once, when the stallion went back to accompany a sick mare (his constant companion) to safety. Piñas took a definite form, unlike the groupings seen in resting.

### 3.2. Pattern Analysis Using Observation Oriented Modeling (OOM)

OOM pattern analysis was used to examine whether the coded behavioral sequences in the four focal event categories (run away, move away, investigation, and stampede) were more consistent with their defined patterns than randomized within-case sequences. Results are summarized below as Percent Correct Classification (PCC) and c-values from 1000 randomized trials. High PCC reflects agreement across all coded elements of the sequence, whereas complete-case classification reflects agreement across the full event sequence and is therefore more stringent.

#### 3.2.1. Run Away Pattern Analysis

**Figure 6:**
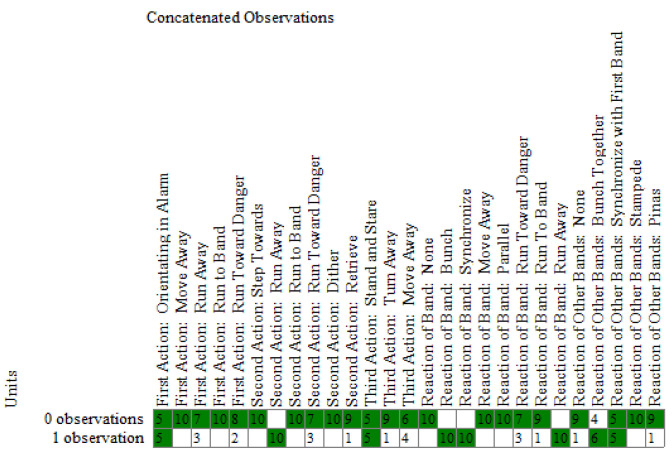
Concatenated observations pattern analysis for *run away*. Each panel shows the concatenated orderings output across 27 binary behavioral orderings. In this figure, units are the two coded observation states for each ordering (0 = behavior absent, 1 = behavior present, within a given event sequence). Green shading indicates observations classified in accordance with the defined pattern, whereas unshaded cells indicate mismatches between observed and expected values. The analysis included 10 complete events (270 observations total), with missing values omitted from totals and 1000 within-case randomization trials (summarized by c-values not included in figure). PCC = 91.1%.

The run away analysis included 270 coded observations derived from 10 complete event sequences (Figure 6). Of these, 246 observations were classified in accordance with the defined pattern, yielding a PCC of 91.1%. In 1000 randomized trials, PCC values ranged from 58.5% to 74.0%, and none equaled or exceeded the observed value (c < 0.001). Complete-case classification accuracy was 20.0% (2/10 cases). These results indicate that the observed run away sequences were more pattern consistent than randomized sequences at the level of coded observations, although substantial variability remained across full event sequences.

#### 3.2.2. Move Away Pattern Analysis

**Figure 7:**
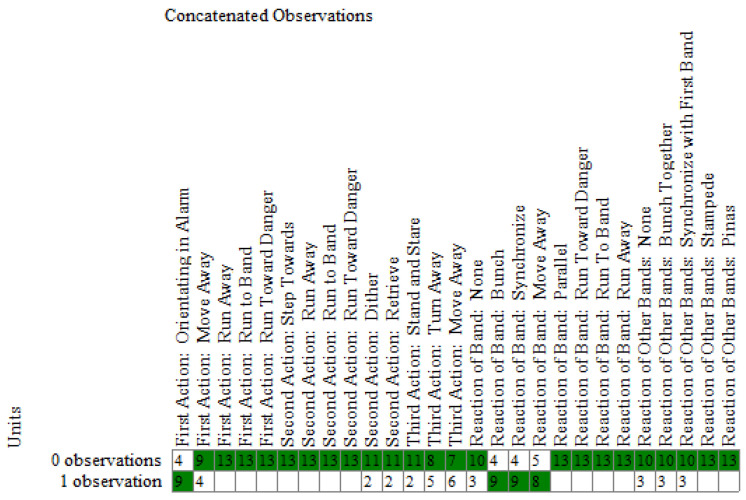
Concatenated observations pattern analysis for move away. The analysis included 13 complete event sequences coded across 27 binary behavioral orderings, yielding 351 total coded observations. Here, units refer to the two coded states within each binary ordering as 0 = behavior absent and 1 = behavior present for a given event sequence. Green shading indicates observations classified in accordance with the defined pattern, whereas unshaded cells indicate mismatches between observed and expected values. Missing values were omitted from totals, and randomization was conducted within cases using 1000 trials (summarized by c-values not included in figure). Observed PCC = 85.8%.

The move away analysis included 351 coded observations derived from 13 complete event sequences (Figure 7). Of these, 301 observations were correctly classified according to the defined pattern, yielding a PCC of 85.8%. In 1000 randomized trials, PCC values ranged from 68.0% to 76.0%, with none equaling or exceeding the observed value (c < 0.001). Complete-case classification accuracy was 15.4% (2/13 cases). These results indicate that the observed move away sequences were more pattern consistent than randomized sequences at the level of coded observations, although full event sequences showed considerable heterogeneity.

#### 3.2.3. Investigation Pattern Analysis

**Figure 8:**
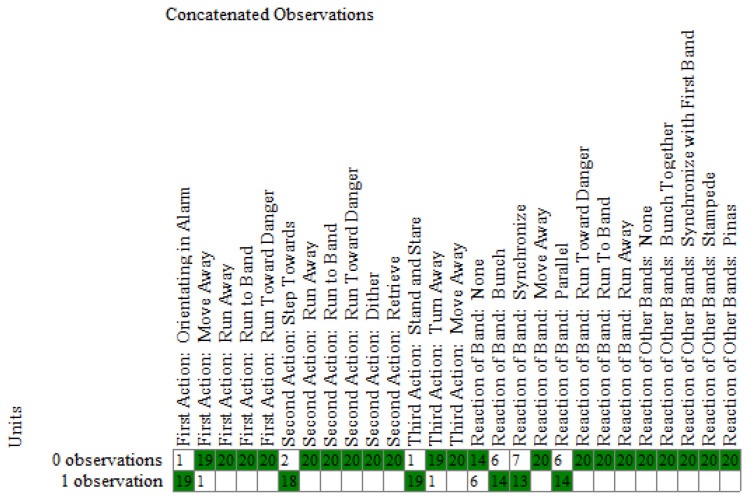
Concatenated observations pattern analysis for investigation. The analysis included 20 complete event sequences coded across 27 binary behavioral orderings, yielding 540 total coded observations. In this figure, units refer to the two coded states within each binary ordering as 0 = behavior absent and 1 = behavior present for a given event sequence. Green shading indicates observations classified in accordance with the defined pattern, whereas unshaded cells indicate mismatches between observed and expected values. Missing values were omitted from totals, and randomization was conducted within cases using 1000 trials (summarized by c-values not included in figure). Observed PCC = 94.3%.

The investigation analysis included 20 complete event sequences and 540 coded observations (Figure 8). Of these, 509 observations were classified according to the defined pattern, producing a PCC of 94.3%. Randomized PCC values ranged from 62.4% to 72.4%, and none equaled or exceeded the observed value (c < 0.001). Complete-case classification accuracy was 60.0% (12/20 cases). These results indicate that the observed investigation sequences were more pattern consistent than randomized sequences, with higher conformity across complete cases than in the other categories.

#### 3.2.4. Stampede Pattern Analysis

**Figure 9:**
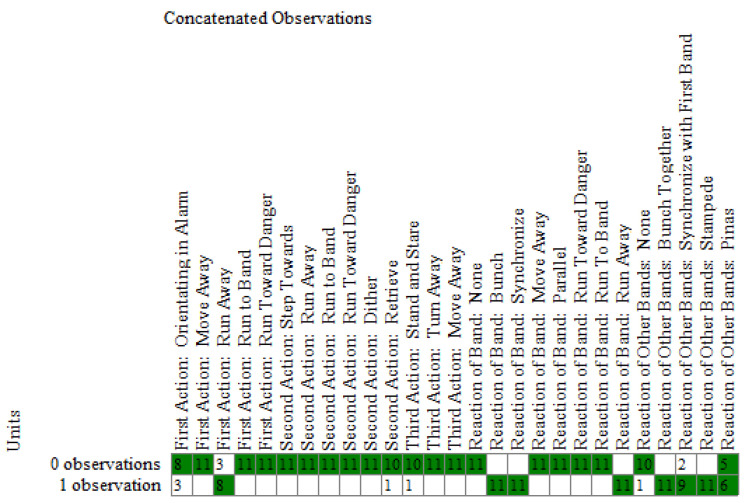
Concatenated observations pattern analysis for stampede. The analysis included 11 complete event sequences coded across 27 binary behavioral orderings, yielding 297 total coded observations. Here, units refer to the two coded states within each binary ordering as 0 = behavior absent and 1 = behavior present for a given event sequence. Green shading indicates observations classified in accordance with the defined pattern, whereas unshaded cells indicate mismatches between observed and expected values. Missing values were omitted from totals, and randomization was conducted within cases using 1000 trials (summarized by c-values not included in figure). Observed PCC = 96.3%.

The stampede analysis included 11 complete event sequences and 297 coded observations (Figure 9). Of these, 286 observations were classified according to the defined pattern, yielding a PCC of 96.3%. Randomized PCC values ranged from 54.2% to 69.4%, and none equaled or exceeded the observed value (c < 0.001). Complete-case classification accuracy was 45.5% (5/11 cases). These results indicate that the observed stampede sequences were more pattern consistent than randomized sequences, while still allowing notable variation across complete event sequences.

#### 3.2.5. Summary of OOM Results

Across all analyses, PCC values ranged from 85.75% (move away) to 96.3% (stampede), with all c-values < 0.001. However, complete-case classification varied substantially across categories, from 15.4% to 60.0%. Taken together, these results suggest recurring structure in the coded event sequences relative to randomized within-case versions, while also indicating that full event sequences were not uniformly captured by the defined patterns.

## 4. Discussion

Large predators are part of the ecological background of the llanos, and the horses observed here lived in an environment in which threat-sensitive behavior may plausibly be shaped by both predator presence and other disturbances. The flexible but recurrent patterns analyzed shed considerable light on the social dynamics of natal bands and herds, emphasizing the stallion’s protective role within a generally self-organizing response system. Detailed responses, including individual differences, have been presented to demonstrate their range. One observational result is that the primary preferred response to alarm is not flight but increased cohesion.

Both the raw observations and the OOM analyses suggest that herd responses to perceived threats in this predator-rich environment were structured but variable, and that the form of group response differed across behavioral contexts. The OOM concatenated orderings analyses showed higher pattern consistency than randomized within-case sequences for all four event categories. However, the degree of conformity at the level of complete cases varied across categories, indicating that although many coded elements of the sequences recurred reliably, full event sequences were not uniform. In this sense, the OOM results are best interpreted as evidence of recurring, context-related behavioral organization rather than fixed social rules. The highest overall PCC values were observed for investigation and stampede, whereas run away and move away showed greater variability across full cases. These differences suggest that coordination may become more stereotyped in some contexts than others, while still allowing substantial variation in how particular events unfold.

In stampede, the herd follows the self-organizing algorithm for mass movement found in other herds, flocks, and shoals [16,17,18]: cohesion, synchrony of direction and velocity, and collision avoidance. Move away and run away show the same features but scaled down; other bands are not recruited. In undisturbed populations, Maeda et al. [38] found a multilevel system of associations, with closely bonded subunits of two or three individuals grouped together in bands, which, in turn, were more loosely associated into herds. Ozogany et al. [39], examining collective movements in Przewalski’s horses, saw “families” of mares and offspring moving together in identifiable bands that traveled as a herd in the same direction. These whole-herd movements represent an even lower level of arousal than those registered in our observations, which were limited to alarm events, but show the same cohesion (proximity), synchrony (“movement similarity”), and collision avoidance (social spacing). In resting huddles, synchrony is confined to (non)activity and social spacing is reduced. The system responds flexibly to physiological arousal.

Preferential proximity is a marker of affiliative bonding [40], as seen in family subunits or friends; it is also used to identify bands, where inter-member distances are larger. In most populations, stallions keep bands separated, so herds do not form. The Camoruco stallions’ herd formation may not be a response to predation. Plains zebra, which have the same basic social organization as horses (natal and bachelor bands, natal dispersal) form similar fission/fusion herds in open savannah, where resources are evenly distributed, whether predators are present or not [41]. A particular influence for the cimarrons is the llanos winters, which bring dirty, knee-deep floodwater and plagues of biting insects for months, which can kill any horse with even small open wounds. Camoruco stallions are blemish free, unlike other feral stallions; they resolve conflicts with subtle signals rather than fights and co-exist peacefully. While band formation is universal amongst horses, suggesting a solution to inherent problems, herd formation is a flexible response depending on environmental context.

Cohesion is the primary response to alarm; its importance is underlined by observations of horses running nearer to a threat in order to reach the safety of others beyond. A startled foal’s run to its mother or an outlying individual’s run to band are directed towards affiliates. The stallion’s centrality in the band’s social network, shown by his affiliation to all its members including foals, makes him the focus of their cohesive movement, as does his particular role as the band’s principal assessor of danger, protector of foals and retriever of straying members.

The Camoruco stallions were notably protective towards foals, the herd’s most vulnerable members, revealing a paternal investment shown in other studies [6,11,42,43]; its importance may be underrated in predator-free zones, where defensive practices may be relaxed [11]. The horse’s trickle-feeding digestive system imposes prolonged grazing times on mares in frugal conditions, while requiring foals to be followers and not hidden like the calves of many ruminants [44]. Though protective towards to newborn foals, mares are less attentive after post-partum estrus when lactation and grazing times increase [39]. Where predators abound, vigilance and foal recovery are necessary and are preferentially undertaken by the stallion, whose maintenance requirements are lower than lactating mares’ [13]. Stable mare–stallion relations help to validate his paternal investment. Stallions vary in their propensity to defend mares or foals; for instance, during the years of study, Jotero maintained his mares closely herded, usually separate from the herd, but was never seen to retrieve a foal, while Amiguete let his mares mingle in the herd but often retrieved foals, or stood over them while resting, even those not his own. Both strategies increase a stallion’s success in terms of surviving foals, and most Camoruco stallions adopt a balance between the two.

Stallion protection may affect mares’ choice where, as in the llanos, resources are uniformly distributed and there are no home ranges. When Careto was lame from a leg wound, his mares were notably loyal to him, but when the resulting tetanus infection crippled him, they abruptly left him. In free-ranging ponies in Galicia, Spain, colt foals are culled, leaving a stallion:mare ratio of 1:20 or more. In wolf-free zones, stallions are accompanied by about four mares, with the remaining mares wandering with their offspring or in pairs; however, where wolves are present, average band size soars to nearly 22 in summer and 12 in winter [45]. Foals less than six months old, i.e., in the summer, are most vulnerable to wolf attack [46], so mares with vulnerable foals ally with stallions despite uncomfortably large band sizes, in which aggression rates are higher [47]. “Loyal” mares have better breeding success than “mavericks” that, wandering from band to band, often lose their foals [11].

The vital importance of a stallion’s correct identification of danger perhaps affects the developmental period that colts spend in bachelor bands [48], when they are free-ranging, adventurous and have a high mortality rate, perhaps due to misadventure [11].

The striking synchrony seen in stampede, echoed in run away and move away, was also seen by [38] in rest/move synchrony of subunits and by Ozogany et al. [39] (direction and velocity). In investigation sequences, colts or resident bachelors lined up beside the stallion and synchronized with his vacillations and standstills. Both colts and resident bachelors have strong affiliative links to the stallion [42], expressed in play and proximity, and their attention to his interests represents not only the synchrony of emotional state for which horses are known [49] but also an opportunity for social learning [43].

The connection between affiliation, proximity and synchrony is complex and poorly understood. Affiliation induces proximity, and proximity induces synchrony, but proximity alone can produce synchrony. In stampedes, bands lose their unity, so that members of different bands run side by side but keep synchrony—as do racehorses whose jockeys have fallen. Small foals, though, synchronize only with their mothers, and stop galloping if proximity is lost, although surrounded by other gallopers. Whether imposed proximity and synchrony promote affiliation, as they do in children [50] and soldiers, amongst others [51] is an unexplored question. Puzzling events are those in which synchrony can have two different effects. In about half of run to band sequences, frightened horses galloping into a calm band alarm the band, but in the other half the band’s tranquility calms them. The same was true of fleeing bands that ran to join calm ones. A trotting horse passing close by a walking one sometimes induced the other to trot, or it might drop to a walk too.

At band level, activity synchrony is a constant feature [52], maintaining band unity in grazing, resting, and marching. Changes in activity are initiated by the most motivated, with there being no specific leaders [53,54]. Thus, marches to water after rest are often led by lactating mares. In ibex, decisions to abandon synchrony with the band’s activities were shown to depend on motivation due to maintenance requirements [55].

Synchrony keeps the fleeing herd together and is observed both front to back and back to front; that is, horses in the middle of the herd may make decisions other than following the leaders and, in doing so, influence the actions of those in front of them. In cattle roundups, the older horses’ experience motivated them not to cross the terraplen, and their avoidance action was transmitted to the front runners. Similarly, in one stampede, one band of bachelors did not partake but watched; it was provoked by a colt that, after socializing with them, took a shortcut back to his band, crashing through bushes. The noise startled all those unaware of its cause, but not the informed. Thus, motivation, sometimes a result of knowledge, can result in initiating changes or failing to synchronize. When all are equally motivated by fear, synchrony is complete.

Collision avoidance is the third necessary element in stampede, when a fall can be fatal. Even small foals cohere and synchronize with their mothers in movement, so these elements are at least partly innate, though a foal’s cohesion to his mother is reinforced by frequent milk rewards; however, young foals seem unaware of the individual space of others [56]. Aggression, usually expressed as threat, frightens an offender out of the aggressor’s immediate vicinity. Intraband aggression has a variety of proximate causes [51,57], but its cumulative effect, or final cause [57,58], is respect for individual space. Notably, more aggressions are directed by adults towards youngsters, suggesting a learning effect. In stampedes, when all are frightened, respect for space is absolute despite close cohesion.

In horses’ undisturbed lives, the repeated themes of cohesion, synchrony, and social spacing suggest that bands are self-organizing defensive cooperatives whose members have different roles according to sex and age, linked together in a network of affiliative bonds that facilitate communication. More generally, self-organized collective movement has been interpreted as permitting flexible group responses, without requiring fixed leaders or rigid social structures [17,18]. The stallion’s role as the band’s protector focuses especially on small foals, increasing his offsprings’ survival rate where predation is a reality. Because lactation imposes substantial energetic demands, it is possible that mares, without the support of a stallion, may face trade-offs between foraging, maintaining condition, and remaining in close association with a foal. Yet despite the differences in motivation arising from differences in role and maintenance requirements, the elements of the massed flight algorithm of cohesion, synchrony, and collision avoidance are a constant undercurrent in band behavior, ready to be upgraded into survival tactics in the face of threat.

An interesting footnote to the revelations this study has provided is the cimarrons’ unusual avoidance of trees. Horses modify their foraging sites and routes to avoid places of known danger, as do other prey animals [42,59]. Pyrexia may have increased the mares’ tendency to seek shade despite the greater apparent predation risk associated with the copses. Avoidance of certain areas may thus be considered cultural: youngsters learn from adults that some areas are off-limits without having experienced why.

This analysis of anti-predator defense behavior, where different roles are paramount, sheds light on many social problems of domestic horses, whose groupings seldom reproduce the security networks of natural bands; moreover, colts seldom enjoy the role model aspect of fathers, and bonds are often broken. These findings may also be relevant to domestic management, because social arrangements that differ from stable natural band structure may influence cohesion, spacing, and conflict. In pastured horses, the lowest aggression rates were found in groups composed of a stallion, mares, and foals [60]. Finally, these observations may be useful to managers of rewilding projects where predators are found. Stallion-led established bands are indicated, preferably with previous experience of predators.

The limitations of this study include observational analysis only (versus video and recorded data) and behavioral coding using standardized software. More than one observer was present at each observation time, so all behaviors were corroborated and confirmed within the group of trained ethologists. Additionally, specific measurements of soil and environmental conditions and predator number were absent. The OOM analyses should also be interpreted as descriptive analyses of the coded event sequences within this dataset; because events were drawn from repeated observations of the same population, they should not be treated as fully independent confirmatory units.

## 5. Conclusions

Observations of feral horses living in a predator-rich environment in the llanos of Venezuela were directed specifically toward alarm, vigilance, and threat response behavior. The horse’s two-level defense strategy consists of precautionary measures, including group living and avoidance of known danger zones, and reactions to perceived threat with assessment, communication of alarm, and massed flight following the behavioral algorithm of cohesion, velocity and direction synchrony, and collision avoidance. The self-organizing nature of the strategy allows widely varied responses. The stallion plays a key though not exclusive role in perceiving and assessing danger. Elements of the same behavioral algorithm are also marked features of maintenance behavior, suggesting that feral horse bands are self-organizing defense cooperatives.

## Figures and Tables

**Table 1:** Ethogram of alarm and subsequent behavior, as well as abbreviations used in data collection and in the following text.

Behavior	Abbreviation
Horse orientates towards threat, ears pricked forward	O
Alarm posture (d, Figure 2); neck fully elevated, high muscular tension [6,10]	A
Bunch together; band members move to increase cohesion	B
Band runs toward and coheres with other band(s)	Bb
Band runs away from perceived threat	BRa
Band runs towards threat to reach other band(s) beyond it	BRt
Horse moves towards threat; takes one or two steps towards threat	Mt
Horse turns and moves away from threat at walk or trot	Ma
Horse runs away at gallop	Ra
Individual runs to join band from outlying position	Rtb
Individual runs towards and past threat to reach band beyond it	Rt
Individual stands staring towards direction of threat, vigilant head position [10]	SS
Stampede, whole herd in flight	St
Individual turns away from perceived threats	Ta
Move to stand in parallel with stallion (young in-band males)	//

**Table 2:** Development of startle/escape events. Inv: investigation; Rtb: run to band; Ma: move away, at a walk or slow trot; Ra: run away, at a canter or gallop; St: stampede, all visible horses running together; Vacillate: move to and fro, without a clear action forthcoming; Bunch: move to cohere or group closely; Synchronize: move together in the same direction and velocity; Retrieve: stallion turns back to retrieve foal or mare.

Class	Inv	Rtb	Ma	Ra	Stam
Numbers	20	23	13	10	11
1. First reaction of startled individual					
OA	19	18	9	5	3
Move away	1	1	4		
Run away				3	8
Run to band		4			
Run towards and past threat to reach band		5		2	
2. Second action					
Step towards	18				
Run away				10	
Run to band		23			
Run towards and past threat to reach band				3	
Vacillate			4		
Retrieve			2	1	1
3. Third action					
Stand stare	19	3	2	5	1
Turn away	1	20	5	1	
Move away			6	4	
4. Reaction of band					
none	6	13	3		
Bunch	14	10	9	10	11
Synchronize	13	2	9	10	11
Move away		2	8		
Parallel	14				
Run towards and past threat to reach other bands				3	
Run to herd				1	
Run away				10	11
5. Reaction of other bands					
none		1	3	1	1 **
Bunch together			3	6	11
Sync with first band			3	5	9 ***
Stampede					11
Piñas				1 *	6
total	20	23	13	10	11

* Band in which stallion retrieved sick mare. ** One band stood and watched (colt in bushes, see Discussion). *** Once, the herd split into two, which ran different ways (loud sound).

## Data Availability

The data supporting the reported results are available in the Supplementary Materials as an Excel sheet of observations. Additional details may be available from the corresponding author upon reasonable request.

## Supplementary Materials

The following supporting information can be downloaded at https://www.mdpi.com/article/10.3390/ani16121826/s1, File S1. Stallions and Bands; Table S1. Natal Bands; Table S2. Supporting Data Defense Tactics.
