# Supplementary material for: Observations on Anti-Predator Defense Behavior in Feral Horses in Venezuela
Source: Animals (Basel). 2026 Jun 12;16(12):1826. doi: 10.3390/ani16121826 (PMC13295745; doi:10.3390/ani16121826)
Supplement: Supplementary file 1 [file animals-16-01826-s001.zip › File S1. Stallions and bands.pdf]

Stallions and bands

TERMS USED HERE AND IN TEXT
Stallion: male consort of natal band
Bachelor: young male not yet with mares
Novice stallion: stallion in his first year with mares. Separated from main herd. Marked *
Mare: breeding female
Maiden mare: mare that appears mature but does not appear to have foaled
J: juvenile, 1-2 years old (possibly 3)
Foal: born that year
Mixed band: bachelors and maiden mares together

Note on difficulties of identification. Fission-fusion of bands was constant. About half the horses were chestnut with some black in the tail. Some had socks but these often could not be seen.
Furia and Humo, both tall, black and elegant (brothers?), could only be distinguished face-on (stars).
Gitana (Gypsy) was identical. She was seen in several different bands.
Guapo and Lazan were both chestnut but Guapo was very slightly darker and had a blaze, Lazan not.
The 3 Nicos also appeared to be identical brothers. Rich dun, with slightly different white facial markings. Nicolás had mares in 2009, Nicordón and Nico Lucero held a band jointly in 2011.
Most stallions had two or three mares that were always with them
Small groups of 2-4 juveniles (1-3 years old, both sexes) often wandered from band to band
Observations began just before dawn 5.30 and stopped around 10.30-11. The light changed considerably during that time, so coat colour appeared different in photos.

2007 Exploratory year, 8 days, no course.
Estimate of population: 140
Skulls: since last flood: ♂♂ 20-25, 6,
*Amiguete in bachelor band of 6, later joined by 1 mare
Bambu 8-9 mares
Careto 7 mares, 4j +2 foals
Dorado 6-7 some mares, some males
Eduardo & Ramon, 2 small bachelor/stallions, 1 mare. Eduardo very thin
Furia: 3 mares, 1j ♂ + 2 foals
Guapo 3 mares, 2j, 2 foals
Humo 8: H, 4 mares, 2 maiden, j ♂ + 2 foals
Ilusion 2 mares, 2j
Jotero 14: J, 9 mares, 3j + 1 foal + donkey
Lazan 3 mares + 1 foal

2008
Amiguete 3-4: A, 2-3 mares, mule foal
Bambu 10: B, 9 mares, 3 foals. Bambu looking stiff, white hairs on cheeks and above tail
Careto 10: C, 8-9 mares, impossible to determine as always in herd, js + 3 (?)foals
Dorado 5: D, 4 mares, 3 foals (+ one mare very very thin, disappeared)
Furia 9: F, 8 mares + 3 foals+ 1 donkey
Guapo 10: G, 7 mares, 2 maidens + 1 foal

Humo 9: H, 9 mares
Jotero 14: J, 9 mares, 4j,+ 3 foals
Orejas and helper (Ramon 2008?): 2 thin mares

2009

143 + 24 foals
2 skulls, both ♀ , 4 y-o and 20-25 y-o
Amiguete 8: A, 4 breeding mares, 2 maidens, j. ♂, + 3 foals + j. mule
*Bambino. 2 mares, 3j ♀
Careto 15: C, 9 mares, 5j, + 4 foals
Dorado 12: D, 5 mares, 4 bachelors, 2j + 4 foals
Elegante with 4-6 bachelors + 2 maiden mares
Furia 3 mares, j♂ + 2 foals
Guapo 4 mares, 2 j ♀ , one of which he ejects from band late April + 1 foal
Humo. 7 mares + 4 foals
Jotero 8: J, 7 mares, + 3 foals
Lazan 5: L, 3 mares + 1 foal
Orejas bachelor this year
*Nicolas 2: N, I mare + foal
*Padrote 3: 1 mare + foal, later 2nd mare
*2 small stallions, 7: 2 mares, 3 j. One ♂ had swollen leg

2010 Very bad drought
153 + 17 foals
Bodies:
Mare 20+, damaged last incisor, points on molars+ dead foal
Mare
5 Foals, two eaten, cranium crushed at back
Body mostly submerged in mud, unidentifiable, near Flor Amarillo. (Careto? If so, tetanus). Age about 14.
Skull ♂, 25+, probably from 2008-9: Bambu?

Bands
Amiguete 8 mares, 3j + 2foals + j.mule
Bambino 6: B, 2 mares, 3j
Careto 14: C, 13 + 2 foals. Careto died, Jotero claimed all his mares but gradually lost many
Dorado: 22 + 4 foals
Elegante, 4 bachelors: 2 mares + 1 foal
Furia: 4-5 mares, 2j +I foal
Guapo 7: G, 3 mares, 3 bachelors, 4j
Humo 8: H, 7 mares + 4 foals
Jotero: up to 20 mares, many from Careto, gradually diminish + 5 foals
Lazan. 8 mares, 3j, + 3 foals
Nicolas: N, 2 mares, 1j
*Nicordón & Nico lucero sometimes with 2 maiden mares.
Orejas + small helper 4: 2 mares
Padrote 4: P, 3 mares + foal
Ramon 5: 3 mares, 2j. 0ne of the mares died

Sevillano, bachelor who acquires and loses mares various times

2011, year of piroplasmosis and mules

110 head = 101 and 9 live foals. Also 2 foals that died + 3 already dead

An extremely difficult year, with many deaths before and during April leaving band networks broken, their members disoriented, and new stallions recruited from the bachelors. Worse, the escaped mules chased, harassed and tried to rape mares, so the whole population was unstable and disturbed. Meetings between stallions were more likely to include agonistic elements than in earlier years.

(The resident young grey mule, born into Amiguete's band, did not behave like the escapees and did not consort with them.)

Bodies

♀ > 17y.o	12.	♂ Ramon 8 y-o	total
12-17 y-o.	2.	Orejas 12-14 y-o	
6-12 y-o.	4.	Pitium. 4 y-o (during)	♀ 20
3-5. y-o.	2.	Mechas. 4 y-o	♂ 6 (2 stallions)
		Palomino 5y-o	foals 3
		Unidentified. 4 y-o	

Two out of a possible 6 stallions' bodies were found.

Stallions disappeared/ letter reused

Elegante, E now Esplendido

Humo

Jotero, J now Joropo

Lazan

Orejas, O now Opaco

Ramon, R now Ron

Amiguete 7 = A, 4 mares, 2 maiden mares, + j. mule born in band

Bambino 4 = B, 2 mares, 1j

*Chocolate 8 = C, 3 mares, 4js + foal that died

Dorado 6 = D, 1 mare, 3 big bachelors, of which 1 died (Pitium), 1 j♂, sometimes joined by 3 young bachelors. D clearly dying at end of April

*Esplendido 5: 3 mares, 1j ♂

Furia 12 = F., 4 mares, 3 maidens, 2 j. ♂, mule, foal

*Joropo 5 = J, 4 bachelors, joined by 1 mare with j. ♂

*Nicos 9 = 2 Nicos ♂ , 4 mares, 1 maiden, 3 j. ♂. Foal that died, his dam emaciated.

Nicolas 8 = N, 3 mares, 4j, + foal. Running battle between N and black mule for possession

*Mixed band 6 = 2 big bachelors, 2 j. bachelors, 2 j. ♀

*Opaco 8 = O, 4 mares, j♂ + 2 foals + one born + one that died

Padrote 10 = P, 5 mares, 3 j. ♂, j. ♀ + 2 foals

*Ron 4 = R, 1 mare, 1 j. ♀ , +j ♀ that died

Bachelors' progress to stallion

Amiguete

2007 with bachelors but at end, joined by grey mare apparently maiden

2008 hovering around but apart from main herd with grey mare, finally joined by 2 more

2009 grey mare has mule foal

Nicolas, no white on face

2009 I mare (Gitana), 1 foal. N looks small and underdeveloped – young??

2010 no mares, mostly in Dorado's band

2011 3 mares

Padrote

2008 in bachelor band

2009 1 mare, then joined by 2nd

2010 3 mares

Elegante

2008 in bachelor band

2009: band joined by 2 young mares but Elegante definitely their stallion

2010: continues with 4 bachelors, 2 mares 1 foal, but ejects 1 bachelor

Nicolas

2008 in Dorado's band

2009 1 mare and foal
