# Supplementary material for: Observations on Anti-Predator Defense Behavior in Feral Horses in Venezuela
Source: Animals (Basel). 2026 Jun 12;16(12):1826. doi: 10.3390/ani16121826 (PMC13295745; doi:10.3390/ani16121826)
Supplement: Supplementary file 1 [file animals-16-01826-s001.zip › Table S1. natal bands.pdf]

	Amig	Bambu	Bambino	Careto	Dorado	Elegante	Furia	Guapo	Humo	Ilusion	Jotero	Lazan	Nicolas	Nicos *	Orejas	Padrote	Ramon	Choco	Espl	Joropo	Opaco	Ron
‘07	bach	10 (**8-9**		12 (**7**)	8 (**4**)	bach	4 (**3**)	6 (**3**)	9 (**6**)	5 (**2**)	14 (**9**)	4 (**3**)			bach		4* (**2**)					
‘08	3-4 (**2-3**)	10 (**9**)	bach	14 (**8-9**)	5 (**4**)	bach	9 (**8**)	10 (**7**)	9 (**8**)	■	14 (**9**)		bach		4 *(**2**)	bach	4 * (**2**)					
‘09	8 (**4**)	■	6 (**2**)	15 (**9**)	12 (**5**)	8 (**2**)	5 (**3**)	7 (**4**)	8 (**7**)	■	8 (**7**)	5 (**3**)	2 (**1**)	bach	bach	2-3 (**1-2**						
‘10	12 (**8**	■	6 (**2**)	14 ■	14-22 (**5**)	8 (**2**)	7-8 (**4-5**)	7 (**3**)	8 (**7**)	■	20- (**17-?**)	12 (**8**)	4 (**2**)	2-4 (**2**)	4* (**2**)	4 (**3**)	5 (**3-2**)	bach	bach	bach	bach	bach
‘11	7 (**6**)	■	4 (**2**)	■	6 (**1**)	■	12(**4**	■	■	■	■	■	8 (**3**)	9 (**5**)	■	10 (**5**)	■	8 (**3**)	5 (**3**)	5 (**1**)	8 (**4**)	4(**1**)

Stallions and their bands 2007 -2011

First figure: total band size excluding foals. Second figure In brackets, bold e.g. **(5):** number of mares.

Band sizes often fluctuated due to fission-fusion. Each stallion had a core group of mares that were always present, but others sometimes stayed in the herd while the main band was separate.

Older stallions (Bambu, Careto, Dorado, Guapo, Humo, Jotero) continued to accumulate mares until they died.

Juveniles formed unstable bands, often mixed, which were sometimes found with natal bands. Others stayed always with their natal band until dispersal.

*Two-stallion bands. “Nicos” were Nicordón and Nico Lucero, almost identical and probably brothers. Others were small or weak stallions that collaborated.

■ Disappeared, presumed dead. Some bodies were found.

Bach: in bachelor band

In 2011 many mares had died; this affected some bands more than others. The mules constantly harassed mares and broke bands up.
